# *Bordetella pertussis* in Adult Pneumonia Patients^1^

**DOI:** 10.3201/eid1104.040822

**Published:** 2005-04

**Authors:** Kirsten A. Beynon, Sheryl A. Young, Richard T.R. Laing, Timothy G. Harrison, Trevor P. Anderson, David R. Murdoch

**Affiliations:** * Canterbury Health Laboratories, Christchurch, New Zealand;; † Christchurch School of Medicine and Health Sciences, Christchurch, New Zealand;; ‡ Health Protection Agency, London, United Kingdom

**Keywords:** letter, Bordetella pertussis, pertussis, whooping cough, pneumonia, adult

**To the Editor:** Although *B. pertussis* infection is well-characterized in children, the epidemiology and clinical spectrum of pertussis in adolescents and adults are less well defined. Instances of pneumonia complicating adult pertussis have been reported (*1**,**2*), yet the role of *B. pertussis* in adult pneumonia has not been rigorously evaluated.

This study searched specifically for evidence of *B. pertussis* infection in 304 adults (>18 years) admitted to Christchurch Hospital (Christchurch, New Zealand) with community-acquired pneumonia from August 1999 to July 2000 (*3*). Nasopharyngeal samples and paired serum samples from these patients were stored and later tested for *B. pertussis* DNA and *B. pertussis* antibodies. Culture for *B. pertussis* was not performed because *B. pertussis* was not part of the original pneumonia study protocol.

Nasopharyngeal samples were centrifuged and tested for *B. pertussis* DNA by using the IS481 hemi-nested polymerase chain reaction (PCR) assay described previously (*4*). Serum samples taken from the patients during acute and convalescent phases of disease were tested for immunoglobulin (Ig)A and IgG antibodies against *B. pertussis* whole cell antigens by using enzyme-linked immunosorbent assay (ELISA) (Pan Bio, Queensland, Australia). All positive serum samples were tested for pertussis toxin (PT) IgG antibodies, the most specific serologic marker for recent *B. pertussis* infection (*5*). This assay, which uses highly purified PT as antigen, has been described in detail elsewhere (*6*).

Of the 304 adults, both acute and convalescent phase serum samples were available from 257 patients, only acute phase samples were available from 46 patients, and no samples were available from 1 patient; nasopharyngeal swabs samples were available for testing for 275 patients. Overall, 8 (3%) patients had definite recent *B. pertussis* infection based on *B. pertussis* DNA in nasopharyngeal samples (8 patients) or elevated levels of anti-PT IgG antibodies (single sample with an anti-PT IgG level >100 EU/mL, or demonstration of a >4-fold rise in anti-PT IgG level) (5 patients). Eighteen (6%) additional patients had evidence of possible recent *B. pertussis* infection based on elevated levels of IgA antibody or demonstrated IgG antibody seroconversion to whole cell lysate *B. pertussis* antigens, but had low levels of anti-PT IgG antibodies. A moderate degree of pertussis existed in the community during the study period, with 4–73 notifications per month in the Christchurch region (population 421,000).

Characteristics of the patients with evidence of recent *B. pertussis* infection are shown in the Table. Other respiratory pathogens identified from the patients with definite recent *B. pertussis* infection were *Streptococcus pneumoniae* (2 patients), *Haemophilus influenzae* (2 patients), respiratory syncytial virus (1 patient), and influenza A virus (1 patient). Respiratory pathogens identified in the group with possible recent *B. pertussis* infection were *S. pneumoniae* (6 patients), *H. influenzae* (2 patients), respiratory syncytial virus (2 patients), influenza A virus (2 patients), *Legionella* *pneumophila* (1 patient), adenovirus (1 patient), and *Pseudomonas aeruginosa* (1 patient). No patients died, but 2 were admitted to the intensive care unit. No clinical or laboratory variables distinguished patients with recent evidence of pertussis from other patients in the study, although the former group had higher proportion of current or ex-smokers (85% vs. 65%; 95% confidence interval for the difference 5%–35%).

**Table:** Characteristics of adults with pneumonia and evidence of recent *Bordetella pertussis* infection

Characteristic	Definite evidence of recent *B. pertussis* infection (n = 8)	Possible evidence of recent *B. pertussis* infection (n = 18)
Median age (range)	68 (37–86)	71 (34–95)
Male:female	4:4	13:5
Current or ex-smokers	6 (75%)	16 (89%)
Median (range) duration of symptoms at admission	8.5 (1–21)	3.5 (1–30)
Presence of cough	8 (100%)	15 (83%)
Sputum production	6 (75%)	7 (39%)
Other respiratory tract pathogens identified	6 (75%)	11 (61%)

This study is the first to systematically search for evidence of *B. pertussis* infection in adults with community-acquired pneumonia. We found evidence of recent *B. pertussis* infection in 3% of adults admitted to the hospital with well-defined pneumonia during a period of increased pertussis activity, and weaker evidence in an additional 6%. In comparison, a community-based study of 122 adults with respiratory tract infections found serologic evidence of *B. pertussis* infection in 7% of the patients (*7*). Other studies have reported that pneumonia complicates ≈4% of *B. pertussis* infections in adults (*1**,**2*), with the disease increasing with age (*1*).

*B. pertussis* infection can be difficult to diagnose, especially if symptoms have been present for many days, and we may have underestimated the number of patients with recent pertussis. However, the combination of PCR and serologic testing is one of the most sensitive approaches for diagnosing pertussis in adolescents and adults (*5*). The nasopharyngeal samples may not have been optimal for PCR testing because they were placed in viral transport media and had already been processed for viral studies. Although the viral transport media was not inhibitory to the PCR, the amount of cellular material may have decreased after this processing.

Our findings indicate that a small proportion of adults admitted to the hospital with pneumonia had evidence of recent *B. pertussis* infection. In these persons, whether *B. pertussis* is a primary or secondary pathogen or an innocent bystander is not clear. Further work is needed to clarify the precise role of *B. pertussis* in developing adult pneumonia, the risk factors for *B. pertussis*–associated pneumonia, and the value of specific *B. pertussis* therapy in this setting. These data will also help inform about the role of pertussis vaccination in adults.
